# Sample Preparation of Atherosclerotic Plaque for SAXS/WAXS Experimentation

**DOI:** 10.1021/acsomega.3c00060

**Published:** 2023-04-04

**Authors:** Rebecca R. Mackley, Steven Huband, Tara L. Schiller

**Affiliations:** † Warwick Medical School, University of Warwick, Coventry, West Midlands CV4 7AL, United Kingdom; ‡ Warwick Manufacturing Group, University of Warwick, Coventry, West Midlands CV4 7AL, United Kingdom; § X-ray Diffraction Facility, Department of Physics, University of Warwick, Coventry, West Midlands CV4 7AL, United Kingdom

## Abstract

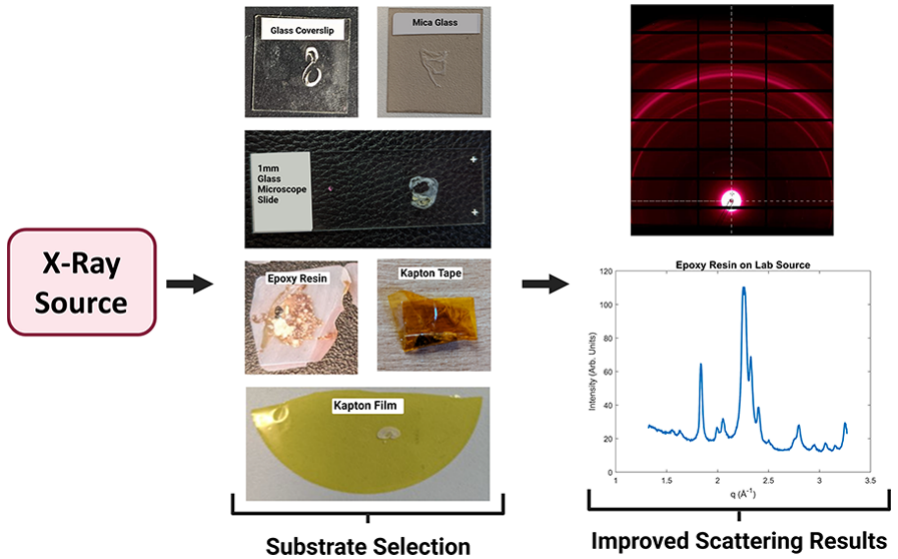

Atherosclerosis is often described as a single disease entity; however, the morphology of each plaque is unique to the individual. The field currently lacks a technique that can discriminate stable from unstable plaques, to identify those at risk of a thromboembolic event. Small- and wide-angle X-ray scattering (SAXS/WAXS) holds the potential to be able to identify key materials present in a plaque, such as cholesterol species, collagen, low-density lipoproteins (LDLs), and hydroxyapatite. Protocols have been established for the preparation of excised human atherosclerotic tissue that are investigated herein. This includes the fixing, sectioning, and substrate selection of the sample. Through several sample preparation methods, vast improvements have been made to sample-to-noise ratio and background subtraction.

## Introduction

In 2019, cardiovascular and cerebrovascular diseases were the first and second causes of death globally,^1^ with most issues being driven by thrombotic complications due to atherosclerosis.^2^ Atherosclerosis is a chronic inflammatory disease characterized by the buildup of an atheromatous plaque along the wall of major arteries. For some, the plaque remains clinically silent. However, for others, the plaque can become unstable and vulnerable to plaque rupture or erosion.^3^ The outcome of this often culminates in a heart attack or stroke.

Atherosclerosis develops over a time span of decades with the first stage often developing during late childhood. It is characterized by lipid deposition in the intimal layer of the artery wall and is associated with inflammation, scarring, and calcification leading to vascular wall thickening, luminal stenosis, and in some cases thrombosis or erosion.^2^ Atherosclerotic plaques can occur in any artery; however, it is most commonly associated with the aorta, coronary, carotid, and peripheral arteries.^4^ Currently, there is no technique available to discriminate stable from unstable plaques to identify those at risk of a thromboembolic event. For a long time, it was believed that stenosis, the degree to which the artery is blocked, was directly linked to the risk of plaque rupture. However, it is now widely accepted that morphology in addition to stenosis is the best predictor of plaque instability.^5,6^

Currently, there are several techniques available to gather data on atherosclerotic plaques, each with their own limitations. Noninvasive techniques include computed tomography coronary angiography (CTCA),^7^ magnetic resonance imaging (MRI),^8^ and positron emission tomography scanning (PET).^9^ Invasive techniques include intravascular ultrasound (IVUS),^10^ optical coherence tomography (OCT),^11^ Raman spectroscopy,^12^ and near-infrared absorption spectroscopy (NIRS).^13^

Techniques such as IVUS and CTCA are routinely used in clinical practice. CTCA is currently the gold standard for noninvasive imaging of atherosclerotic plaque, particularly in the coronary arteries.^7^ IVUS is an invasive imaging technique and was one of the first able to distinguish morphological features of plaques.^14^

There exists an opportunity to develop an imaging technique or device that would be able to discriminate between stable and unstable plaques at multiple locations. Prior to this development, there needs to be extensive chemical, structural, and spatial information available on the different plaque components to define an unstable plaque.

X-ray scattering is an analytical technique in which X-rays are deflected and scattered by a sample producing complex patterns. Analysis of these patterns can be used to determine the size, shape, and structural features of the sample.^15^ The use of SAXS and WAXS with atherosclerosis is a relatively new technique. Currently, it has been used to model the structure of LDLs and investigate the orientation of collagen fibrils.^16,17^

For SAXS and WAXS analyses, the intensities of X-rays scattered by a material are measured as a function of the scattering angle. This is applicable to both crystalline and amorphous materials. SAXS allows for the diffraction patterns of larger structures common in proteins to be investigated. WAXS or X-ray diffraction is a technique that provides information on the atomic structure of materials. The positions and intensities of peaks in a diffraction pattern are related to their atomic structures. This makes X-ray diffraction a powerful technique for identifying phases contained in samples.

When X-rays interact with a sample, they are scattered or deflected by the material. This scatter is then collected and processed to produce 1D diffraction plots containing peaks. Using reference databases and measuring standards, peaks are identified as being suggestive of materials. To fully confirm this identification, spectroscopic analysis would be required. The scattering angle of a diffraction peak is given by Bragg’s law. Often the scattering vector q is used to represent scattering data as it is wavelength-independent (*q* = (4π/λ)sin θ). Through Bragg’s law, the relationship between *q* and the *d*-spacing is given by *q* = 2π*n*/*d*.^18^

The data ultimately collected will be of diffraction peaks and diffraction intensity, which will be used to suggest the identification of materials associated with the samples. No form factor analysis was performed.

Using both SAXS and WAXS allows the structures of materials to be measured from the interatomic distance up to the larger length scales of proteins. A method has therefore been developed to optimize diffraction data for human atherosclerotic plaque.

## Experimental Section

### Carotid Plaque Samples

#### Carotid Endarterectomy

**Figure 1:**
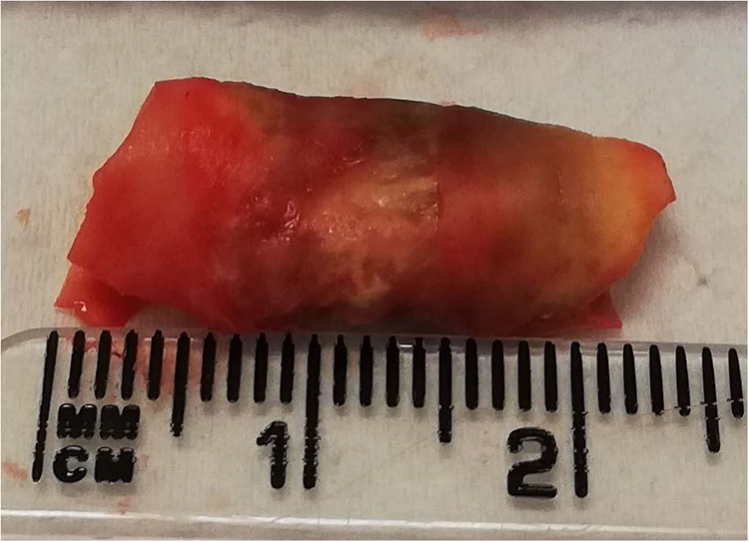
Excised carotid plaque. Black areas of the plaque represent necrosis, whereas the more yellow areas represent lipid-based materials.

A carotid endarterectomy (CEA) is the routine procedure in which atherosclerotic plaque is removed from the carotid artery of live patients.^19^Figure 1 is an example of an excised carotid plaque with clear areas of necrosis and lipid deposition.

#### Sample Collection

Samples were collected from patients who presented to the Alfred Hospital in Melbourne, Australia, with clinical indications for a carotid endarterectomy. Following on, samples were collected by Arden Tissue Bank at University Hospital Coventry and Warwickshire (UHCW). Patients presented with clinical indications or were recommended for elective surgery.

Carotid plaques were collected in both Australia and England with approved ethics and informed consent. The method development process involved fixing, embedding, and sectioning the samples, then mounting them onto multiple substrates.

#### Plaque Tissue Fixation

The fixing of tissue is an essential step to preserve the cells and tissue components of the sample to obtain reliable results.^20^ The two fixative methods used in this work were 10% neutral buffered formalin (NBF) and glutaraldehyde. 10% NBF is one of the most common fixatives used for light microscopy and glutaraldehyde is commonly used for electron microscopy. The use of fixatives also makes tissues safer to handle due to it being a sterilant that effectively kills microorganisms including viruses.^21^

### Embedding and Sectioning of the Carotid Plaques

#### Paraffin Wax

Routinely for use in histopathology and following formalin fixation, tissues are embedded in paraffin wax. This is referred to as the formalin fix paraffin embed (FFPE) protocol. Once the wax has penetrated the tissue, it is formed into a block which can be clamped onto a microtome for sectioning. When using a microtome, standard sections are between 3 and 7 μm with a maximum thickness often being about 10 μm. Sections are cut as a ribbon, separated, and mounted onto a substrate.

#### Epoxy Resin

Epoxy resin embedding is often used with glutaraldehyde fixation for use in electron microscopy.^22^ A protocol was devised to embed and section large plaque samples in epoxy resin (Merck U.K.). Samples were then sectioned to 100 μm using an IsoMet Precision Cutter (Buehler, Coventry U.K.).

### Substrate Selection

For this work, the substrates can be split into either being glass or Kapton-based. The epoxy resin samples are freestanding, and the resin therefore acts as an embedding medium and a substrate.

#### Glass Substrates

Three glass substrates were used, microscope slides, zero-thickness coverslips, and mica glass. Most often, particularly for use in histopathology or microscopy, FFPE ribbons are mounted onto a standard 1 mm white glass microscope slide. Zero-thickness coverslips are thin pieces of glass, typically 0.08–0.13 mm, and are used to keep specimens pressed flat on a microscope slide. Finally, muscovite mica can be thinly cleaved, typically 0.15 mm, into a square sheet like the coverslips. Muscovite mica is often used as a window in X-ray scattering instrumentation^23^ and as an alternative to glass in high-temperature applications, such as windows in furnaces.^24^

The samples used at the Australian Synchrotron were those collected from the Alfred hospital. These samples followed the FFPE protocol and were sectioned and mounted on 1 mm glass microscope slides. For the coverslip and mica glass, samples were supplied from UHCW. The FFPE protocol was followed and sectioned at 10 μm.

#### Kapton

Kapton is available in two forms, tape or film. The film (Fisher Scientific) is 7 μm thick, and the FFPE protocol was followed to prepare the samples, Whereas the tape (Fisher Scientific) is 100 μm thick, and in this case, the sample was fixed in formalin and then sectioned using a heavy-duty scalpel. The samples are sectioned into ∼1 to 2 mm pieces. If required for experimentation, the sample is placed between Kapton tape. Otherwise, if not immediately required, it is stored in phosphate-buffered saline (PBS) to prevent the sample from drying out. Once mounted in tape, calipers are used to measure the thickness of the sample, which is needed for corrections during processing of the SAXS/WAXS data.

### SAXS/WAXS Instrumentation

Details relating to the experimental instrumentation are summarized in Table 1.

**Table 1:** Details Pertaining to the Analytical Equipment Used for the Samples^a^

name	X-ray source	wavelength (Å)	*q*-range (Å^–**1**^)	SAXS or WAXS
Australian Synchrotron	synchrotron radiation	1.03	0.0015–3 (SAXS) 0.6–5^b^ (WAXS)	both
Warwick Xuess 2.0	Cu-kα	1.56	0.25–3	both
Diamond B21	synchrotron radiation	1.00	0.0031–0.38	SAXS
Diamond DL-SAXS	Excillium Gallium MetalJet	1.34	0.0015–4.9^c^	both

a Including the location, X-ray source, wavelength, and whether it was for SAXS or WAXS. The *q*-range listed in the table is the full *q*-range possible on the equipment. However, this can depend on the scattering power of the sample, the sample-to-detector distance, and the position of the WAXS detector.

b Energy-dependent.

c Depending on the type of sample and detector location.

#### Lab-Based Sources

A 5 m Xenocs Xeuss 2.0 SAXS equipped with dual microfocus (copper/molybdenum) sources and a Pilatus 300 K hybrid photon counting detector at the University of Warwick X-ray diffraction facility was used. Using this equipment, scattering data were collected for samples mounted on glass coverslips, mica glass, Kapton film, epoxy resin, and Kapton tape.

A Xenocs Xeuss 3.0 with both Excillium Gallium MetalJet and molybdenum microfocus sources equipped with an Eiger R 1M detector at Diamond Light Source (DLS), Didcot U.K., Diamond Leeds SAXS facility was used. Samples embedded in epoxy resin and Kapton tape were used on this equipment. For both SAXS and WAXS measurements, the Excillium Gallium MetalJet source with a wavelength of 1.34 Å was used. The detector was placed at 4.5 m for SAXS and 1 m for WAXS measurements. For both lab-based sources, silver behenate was used for calibration.

#### Synchrotron Sources

SAXS and WAXS measurements of the 1 mm glass microscope slide samples were taken at the Australian Synchrotron on the SAXS/WAXS beamline. Experimentation on the resin samples was undertaken at DLS on beamline B21.

## Results and Discussion

After each experiment, the fixation, embedding, sectioning, and substrate were all assessed. Scatter was collected from seven different experiments on four pieces of SAXS/WAXS instrumentation including both synchrotron and lab-based sources. The experiments included line scans or mapping of the sample to investigate multiple areas of the sample and visualize the morphological variability of the sample. An example of the patterns produced from a line scan is shown in Figure S2 (Supporting Information). A summary of the experimental procedure can be found in Table S1 (Supporting Information).

During the first experiment at the Australian Synchrotron, line scans of the samples were taken to assess radiation damage to the tissue. No visual damage was seen, and scatter was collected multiple times at the same location to investigate any differences in peaks present and peak intensity. No differences were found. Samples have also been visualized under a microscope to look for damage, but nothing was found. It has been noted however that over time the samples can dry out.

### Background Reduction and Sample Thickness

To allow transmission in SAXS/WAXS measurements, the plaque samples need to be mounted on a substrate to keep them stable during the measurements and allow them to be translated so different areas can be probed. The material structure of the substrate is important as this will also scatter X-rays and contribute to the background of the 1D diffraction plot. In addition to the structure of the substrate, another important factor to consider is the ratio of the sample to substrate thickness. As the substrate also influences the X-rays, the sample needs to be of sufficient thickness to allow for scattering intensity to produce discernable peaks. The larger the effect the substrate has on the X-rays, the thicker the sample will need to be. The thickness of the sample, however, is often determined by the embedding medium, e.g., it is difficult to obtain samples thicker than 10 μm for FFPE. For some scattering equipment, there is a minimum thickness required to be able to achieve reasonable data. The thinner the sample, the more data collected on the plaques resulting in a higher resolution of the plaque structure. However, if the sample is too thin, it is possible for the substrate to dominate the scattering pattern or for the sample to not scatter the X-rays at all.

**Figure 2:**
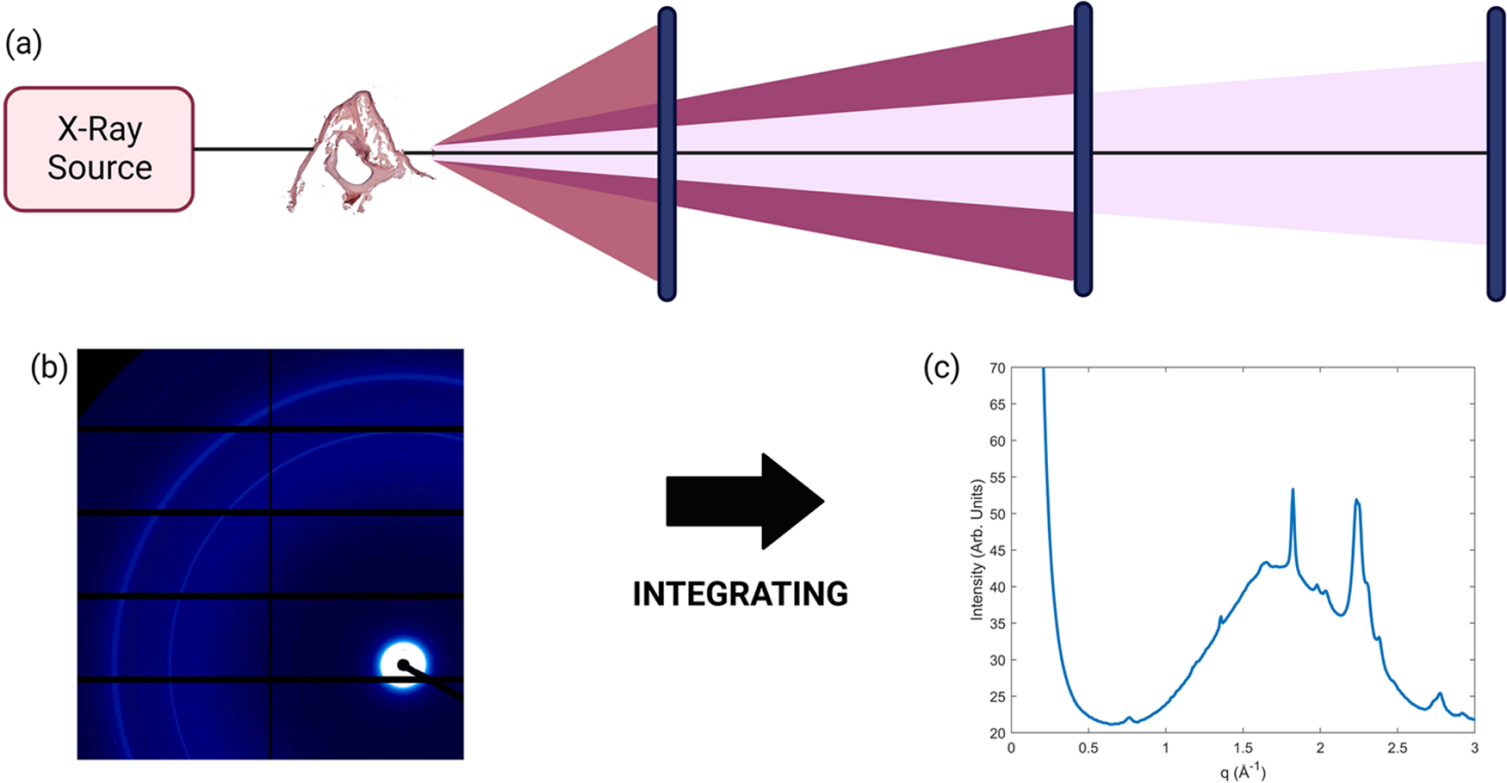
SAXS/WAXS Overview. (a) Schematic overview of the SAXS/WAXS process. An X-ray source scatters off a sample, and that scatter is collected at a detector. Moving the detector further away from the sample allows for the small angles to spread out, making them easier to detect. (b) Example of a detector image from the Australian Synchrotron. Integrating across this detector image produces a 1D diffraction plot (c). The peaks in the diffraction plot equate to the concentric rings seen in (b).

**Figure 3:**
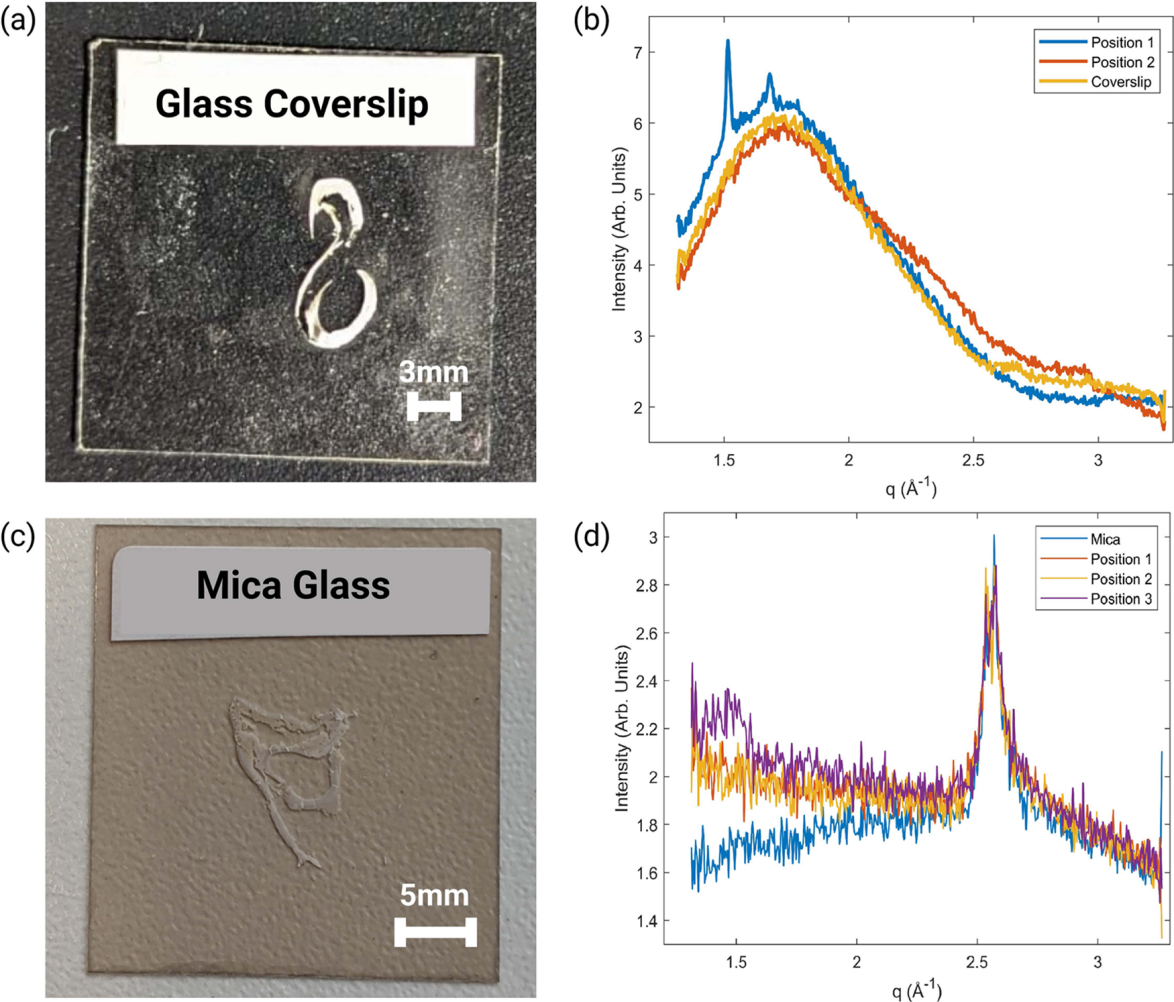
(a) 10 μm FFPE sample mounted on zero-thickness coverslip. (b) The corresponding 1D diffraction plot for the coverslip. The glass hump is still clearly present. (c) 10 μm FFPE sample mounted on mica glass. (d) The corresponding diffraction plot for mica glass. No difference was found when moving from the glass to the sample.

The first experiment was performed at the Australian Synchrotron on the samples mounted on microscope slides. A schematic diagram of the setup is shown in Figure 2. As shown in Figure 2c, peaks present were indicatory of the crystalline domains of hydroxyapatite (Inorganic Crystal Structure Database collection code: 26204). However, it was clear that the scattering pattern was dominated by the glass, which is shown as a broad amorphous peak in Figure 2c. These results would be almost impossible to replicate on a lab-based source as the scattering intensity of the sample would be too low to see any peaks. To reduce the burden of the broad amorphous peak, it was proposed that zero-thickness coverslips could be used (Figure 3a).^25^ While the glass would still produce an amorphous peak, this burden could be reduced. The sample-to-substrate ratio would also be higher, meaning that the signal-to-noise ratio would improve. While these samples produced peaks on lab-based source (Xeuss 2.0 Warwick), the amorphous peak from the glass still dominated the scattering pattern. Peaks present in the scattering pattern were suggestive of materials such as hydroxyapatite (ICSD collection code: 26204) and cholesterol species,^26−28^ as shown in Figure 3b. Results could be improved using a synchrotron source as the power, and tunability should improve the visibility of sample peaks. However, one problem was the coverslips were extremely thin and prone to breaking.

The next substrate trialed on the Xeuss 2.0 was muscovite mica (Figure 3c), which has distinct sharp diffraction peaks rather than a broad amorphous peak.^29^ While this would shroud any material information located at those positions, it is preferable over an amorphous peak. Unfortunately, on the lab-based source, no peaks from the sample were observed. There was no drop in intensity when moving from the mica to the sample. This could mean that the mica is absorbing most X-rays, which in addition to the thin sample, produced no relevant peaks. The only peak found was that of an extreme intensity from the mica itself (Figure 3d). This peak however disappeared depending on the orientation. Therefore, if this substrate were to be used for multiple samples, we would have to ensure that the sample was mounted on the mica at the same orientation. Note the mica is also prone to breaking.

The samples were then once again prepared following the same protocol and mounted on 7 μm Kapton film. Kapton is regularly used in X-ray scattering experiments due to its high thermal and mechanical stability and high transmittance to X-rays,^30^ thus reducing the burden of the substrate background contribution. Scatter was collected on the Xeuss 2.0 lab source and analyzed producing 1D diffraction plots. These plots contained more peaks, of similar intensity to the coverslip samples with the addition of no amorphous peak from the glass. These results were promising; however, due to the thickness of the film, it was very difficult to handle. The thickness of the film also made it very difficult to mount any section larger than 10 μm. Kapton film was shown to be a promising substrate; however, the samples were deemed too thin to produce scattering peaks with enough intensity to analyze.

**Figure 4:**
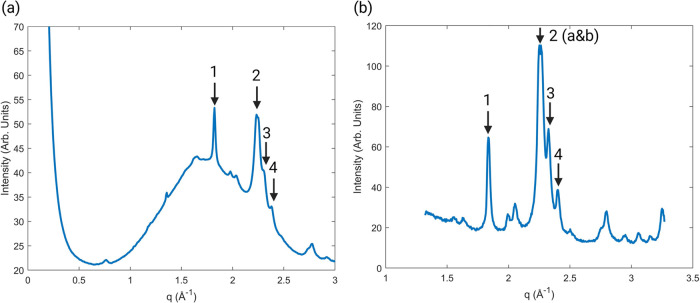
Comparison between hydroxyapatite peaks in samples depending on the source and the substrate. (a) An FFPE sample mounted on a 1 mm glass microscope slide with a synchrotron source. (b) Epoxy resin sample on XENOCS 2.0. The peaks are more clearly resolved in this sample, and the amorphous hump is not present.

During sample preparation, it was noted that due to the presence of hydroxyapatite, the samples were extremely difficult to section. The blade dulls very quickly and often cannot make it through the whole sample. The hydroxyapatite could either be removed or softened with acid, which could produce calcium salts. These could then be mistaken as being inherent to the plaque. It was concluded that another method of fixation and sample preparation was required that would allow both thicker samples and the hydroxyapatite to remain within the CEA sections. Previously, teeth have been sectioned by embedding the tooth in epoxy resin and sectioning using a low-speed cutting machine under a water coolant.^31^ Hydroxyapatite is the main mineral in the enamel of teeth,^32^ and as it is also found in atherosclerotic plaques, this suggests this method would be suitable for sectioning of the plaques. Samples were fixed in glutaraldehyde and embedded in epoxy resin. No substrate was required as the samples were now freestanding in the epoxy resin, and thicker samples were possible. The calcification in the samples could now be seen by the eye (Supplementary Figure 1). On Xeuss 2.0, these samples showed hydroxyapatite peaks with a vast reduction in background contribution (Figure 4b). Figure 4 shows how simply changing the sample preparation method can influence the scattering patterns produced. Except for hydroxyapatite, no other materials were identified in the WAXS of the resin samples on Xeuss 2.0.

#### DLS B21

The lab-based equipment at Warwick was an effective way to investigate the epoxy resin samples using WAXS; however, not much was seen in the SAXS. Therefore, SAXS measurements were taken of these samples on B21 at DLS. The result of this experiment displayed the first possible identification of peaks suggestive of phospholipids^33^ and cholesteryl esters.^27,28^ These were, however, of low intensity. It is possible that the epoxy resin is absorbing a large quantity of X-rays. In addition, during the fixation process, a large majority of the lipids could be stripped away. It is possible to add osmium tetroxide during the fixation process to aid in lipid preservation. Note that great care should be taken as osmium tetroxide is extremely toxic.

It was concluded that the epoxy resin samples are an efficient way to investigate the calcium salts present in the plaque, but not for the lipid-based materials.

#### DLS DL-SAXS

While these results were promising, a conclusion was beginning to form that to achieve peaks in the scattering data, thicker samples were required (>50 μm), which is difficult when sectioning through calcium-based materials. From these experiments, it was concluded that Kapton was a superior substrate over glass.

**Figure 5:**
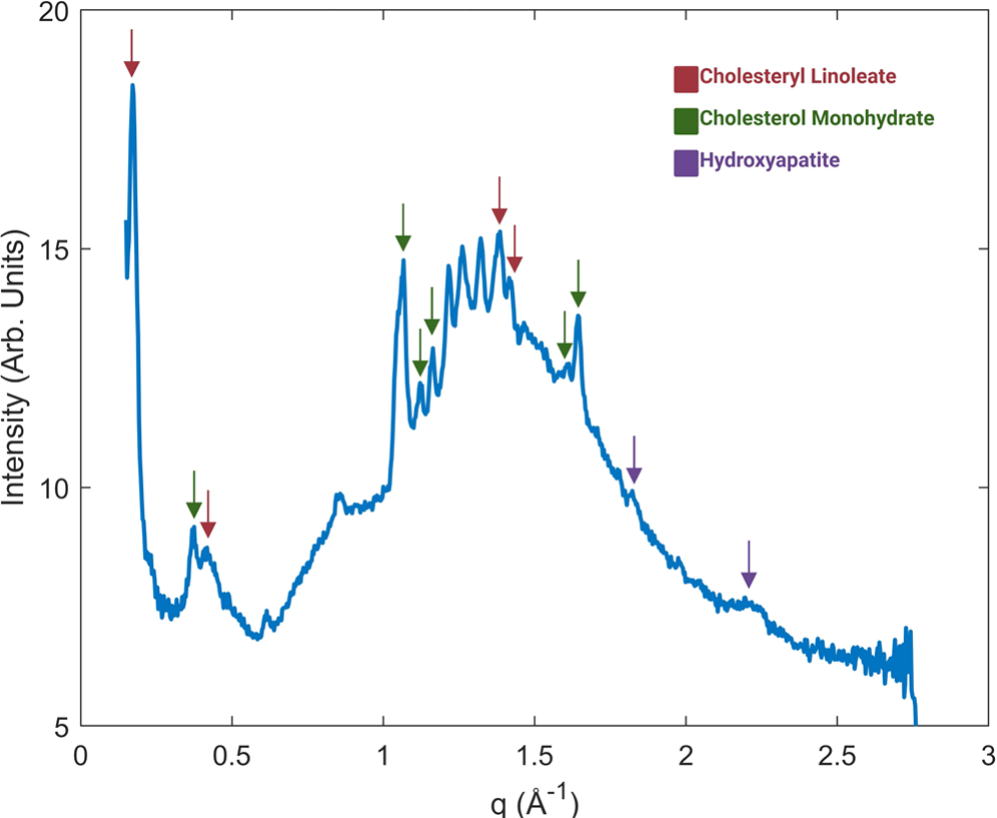
Example of a diffraction plot from a sample sectioned with a heavy-duty scalpel to approx. 1 mm and placed in Kapton tape on a lab-based source. There are more peaks present than any other substrate. The large broad hump present is characteristic of a cholesteryl ester in a liquid-crystal state, which was missed when using a glass substrate.

It was therefore proposed to fix the samples and remove the embedding medium. Kapton tape, similar to the film, has a high transmittance to X-rays and would contribute minimally to the background. The most promising results thus far were gathered on the Xeuss 3.0 at DLS on the DL-SAXS. In addition to the peaks already seen in previous experimentation such as those indicative of hydroxyapatite and phospholipids, other cholesterol-based compound peaks were present, with consistent scattering intensity. Peaks suggestive of cholesterol monohydrate^26,34,35^ were present, along with cholesteryl linoleate.^28^ In addition, an amorphous peak was present around 1.2 Å^–1^, which is not from any glass material (Figure 5). It is believed that this amorphous peak is characteristic of cholesteryl linoleate or oleate being present in a liquid-crystal form.^27,28^ This phase identification would have otherwise been misassigned when using a glass-based substrate.

#### Sample Absorption

The samples used throughout this work were all of varying thickness, with the conclusion that thicker samples provide better scattering results up to a point. It has also been mentioned that some substrates and samples are limited by thickness. However, an important question arises as to whether this limitation would still be applicable if all sample preparation methods were able to produce samples of the same thickness. In this case, variations in absorbance would likely correlate with calcium-rich areas. It is believed that investigating these variations would not be of value add to the research aim of identifying morphological features of a plaque. X-ray fluorescence would be a good alternative technique to give elemental information.

#### Applications

While this technique is far from clinical application, it has the potential to help characterize and define a vulnerable atherosclerotic plaque. Using this technique to map samples would provide the potential co-location of materials. Correlating these materials with patient characteristics such as symptomatic patients could possibly lead to the identification of materials associated with vulnerable plaques.

In addition, the presence of cholesterol monohydrate has been shown to harden the plaque, whereas cholesteryl esters soften the plaque.^36^ This technique would allow insight into the structural build of the plaque.

## Conclusions

Collecting scattering results for solid biological materials can be very difficult as they often require detailed sample preparation. Atherosclerotic plaques are no different, there is no perfect way to prepare these samples. However, there are ways to optimize the samples to get the best results. From this work, the best sample preparation method to study the plaques was through formalin fixation, section with a heavy-duty scalpel, and secure in Kapton tape. Formalin was chosen over glutaraldehyde due to the toxicity of osmium tetroxide required for lipid preservation and for the time required to penetrate the sample. Glutaraldehyde is not suitable for large samples over 1 mm, which is sometimes the case here. While this method is difficult for other complimentary characterization methods, it would be possible to process the sample after experimentation. For example, in most microscopy techniques, the sample needs to be flat for imaging of the surface, which is not the case for this sample preparation method.

This method allows for the study of both lipid-based materials and crystalline materials. Care does need to be taken for the thickness of the sample. If the sample is too thick, then the X-rays cannot fully penetrate the sample.

Table S2 summarizes all of the substrates used alongside their pros and cons for use in SAXS and WAXS experiments.

## Supporting Information Available

Tabulated summary of all seven experiments, tabulated pro and con list of substrates used, detailed image of epoxy resin samples, and graphical representation of a line scan (PDF)

## Notes

Project number 130/11, approved by The Alfred Hospital Ethics Committee.

## Notes

Collection of excised plaques from the carotid artery, 18/SC/0180, approved by Arden Tissue Bank.

## Notes

All figures and images are courtesy of R.M.
