# Supplementary material for: Sample Preparation of Atherosclerotic Plaque for SAXS/WAXS Experimentation
Source: ACS Omega. 2023 Apr 4;8(15):13833–9. doi: 10.1021/acsomega.3c00060 (PMC10116636; doi:10.1021/acsomega.3c00060)
Supplement: Supplementary file 1 — ao3c00060_si_001.pdf [file ao3c00060_si_001.pdf]

Supplementary Information

Sample Preparation of Atherosclerotic Plaque for SAXS/WAXS Experimentation

Rebecca R. Mackley[a,b], Steven Huband[c], and Tara L. Schiller[b]

[a]Warwick Medical School, University of Warwick, Coventry, West Midlands, CV4 7AL, United Kingdom

[b]Warwick Manufacturing Group, University of Warwick, Coventry, West Midlands, CV4 7AL, United Kingdom

[c]X-ray Diffraction Facility, Department of Physics, University of Warwick, Coventry, West Midlands, CV4 7AL, United Kingdom

E-mail: t.l.schiller@warwick.ac.uk

Table S1 – Summary of the seven SAXS/WAXS experiments preformed including the year, location, preparation method and results.

Machine	Fixation	Sectioning	Substrate	Line Scan or Mapping	Reasoning	Results
Australian Synchrotron	FFPE	Paraffin microtome	1mm microscope slide	Line	First experiment to be performed on these plaques.	Good scattering intensity however signal-to-noise hampered by amorphous SiO_2 (glass) broad peak.
Warwick	FFPE	Paraffin microtome sectioned between 7-10μm	Zero thickness coverslips	Line	Less glass to scatter x-rays which would reduce the burden of the amorphous hump.	Extremely fragile substrate and results still exhibit broad SiO_2 peak.
Warwick	FFPE	Paraffin microtome sectioned between 7-10μm	Mica glass coverslips	Line	Remove the burden of the amorphous hump. Mica glass thought to contribute little to the background.	No signal from sample found. Only a single intense peak from the mica. Mica appears to be absorbing x-rays.
Warwick	FFPE	Paraffin microtome sectioned between 7-10μm	Kapton film	Line	Kapton has high transmittance to x-rays	Appearance of some peaks however sample too thin for signal to be discernible.
Warwick	Glutaraldehyde	Diamond-edged saw sectioned to 100μm	Epoxy resin	Line	FFPE protocol believed to strip away calcium salts. Hydroxyapatite difficult to section through using normal microtome	Peak identification of hydroxyapatite in WAXS with improved background.
Diamond B21	Glutaraldehyde	Diamond-edged saw sectioned to 100μm	Epoxy resin	Both	Investigate SAXS of epoxy resin samples	Signal-to-noise low however clear identification of phospholipids.
DL-SAXS Diamond	10% Neutral Buffered Formalin	Heavy duty surgical scalpel	Kapton tape	Both	Thicker sample leading to higher scattering intensity.	Clear identification of cholesterol monohydrate peaks. In addition, cholesteryl ester peaks identified. Amorphous hump from SiO2 not present however an amorphous hump is still clear, which led to the identification of cholesteryl esters in different phases.

Table S2 – Summary of all of the substrates used and a list of their pros and cons. Focusing on both their ability to preform for SAXS/WAXS experiments and also for other complimentary characterization methods.

Substrate	Pros	Cons
1mm glass microscope slide	Fits into most microscopes thus easy for mounting and staining.	Thick piece of glass which scatters x-rays causing a broad peak that shrouds material information. Sample requires embedding before sectioning and mounting.
Zero thickness coverslip	Less glass to scatter x-rays which reduces the broad peak.	Snap very easily, difficult to mount sample. Coverslip still scatters the x-rays. Sample requires embedding before sectioning and mounting.
Mica glass	Limited contribution to x-ray scattering background.	Only one peak seen which is inherent to the mica glass. This an intense sharp peak. No peaks seen from plaques.
Kapton film	Good transparency to x-rays thus limited interference. Insensitive to radiation damage. High thermal stability range.	Extremely thin (~7μm), surface tension dominates making the film difficult to handle and mount sample. Wrinkling of the sample occurs. Sample requires embedding before sectioning and mounting. Would be unsuitable for thick (>50μm) sample.
Epoxy resin	No substrate required. Can be sectioned at 100μm. Calcium deposits readily seen, no need to be removed or softened. Can be imaged using SEM with no coating.	Epoxy absorbs x-rays resulting in low intensity peaks. Charging occurs during other fluorescence imaging resulting in low quality images.
Kapton tape	Good transparency to x-rays, limited interference. Insensitive to radiation damage. High thermal stability range. Thicker than Kapton film.	Still produces a background with peaks for SAXS/WAXS measurements. Sample would need to be processed for any further characterisation.

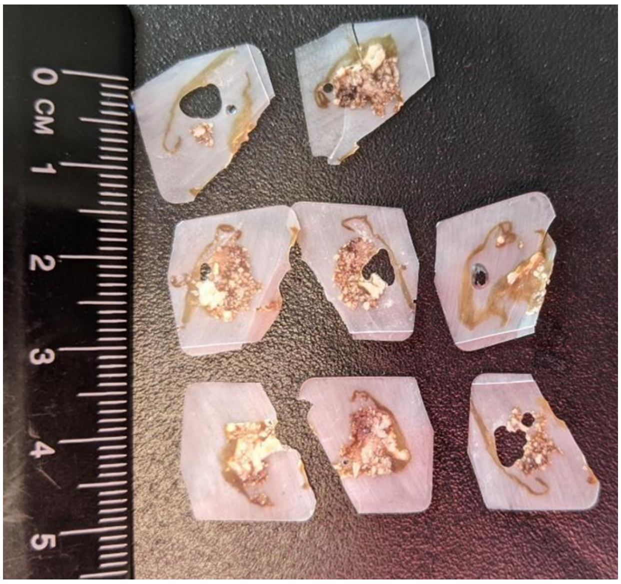

Figure S1 - Epoxy resin plaque samples sectioned at 100µm. Calcification of the sample is clearly visible as the white areas within the brown tissue areas.

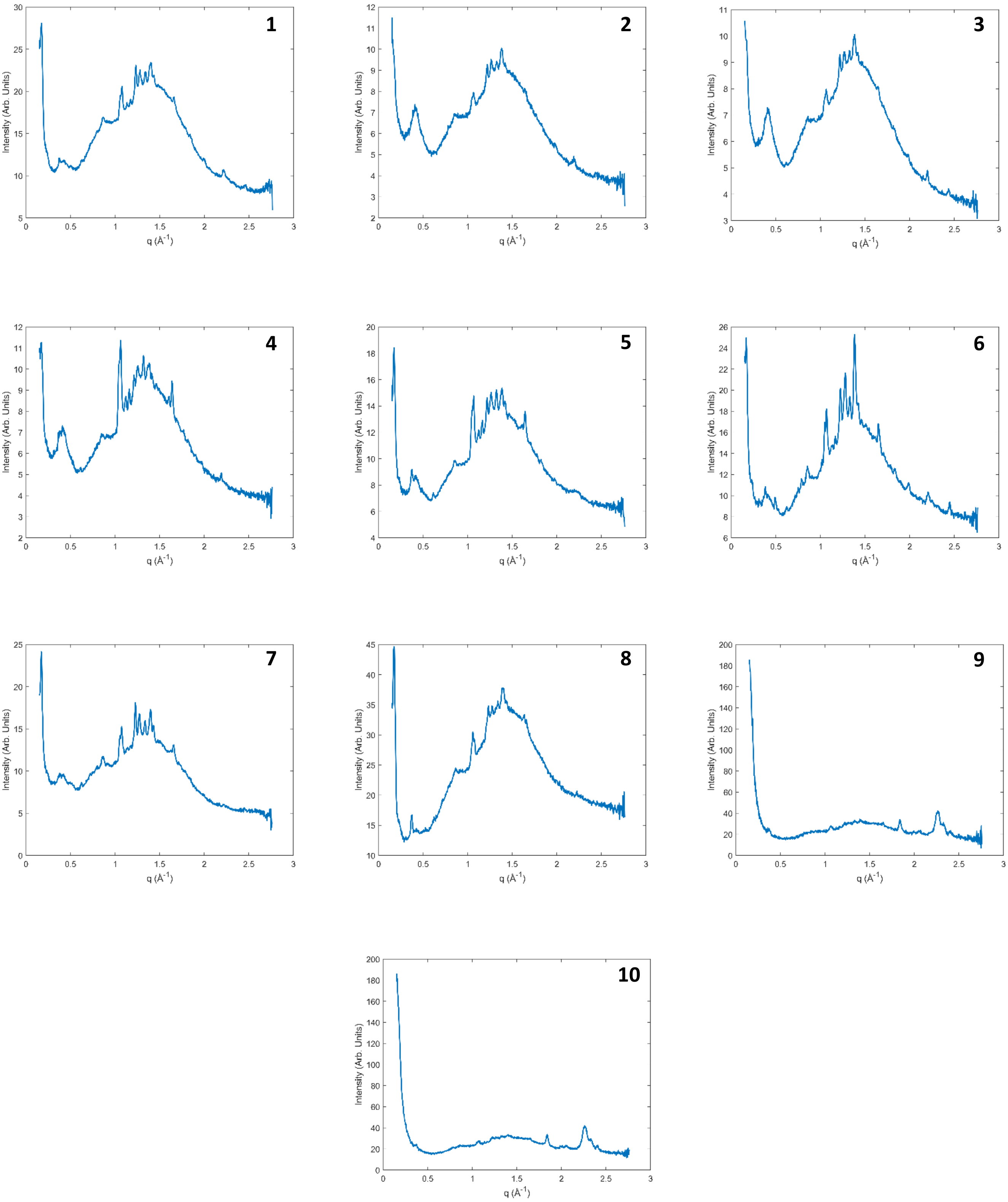

Figure S2 – An example of the graphical output from a line scan of a plaque sample in Kapton tape. The peaks present in locations 1-8 contain peaks suggestive of cholesterol species such as cholesterol monohydrate and cholesteryl linoleate. Then moving to locations 9 and 10, the peaks are suggestive of hydroxyapatite.
